# Do theory of planned behaviour constructs change during adolescent alcohol use onset?

**DOI:** 10.1186/s13011-026-00726-5

**Published:** 2026-04-10

**Authors:** Richard Cooke, Geraldine Mabille, Frode Adolfsen, Roman Koposov, Henriette Kyrrestad

**Affiliations:** 1 School of Health, Education, Policing & Sciences, Department of Psychology, University of Staffordshire, Stoke-on-Trent, UK; 2 Department of Psychology, University of Nottingham, Notthingham, England, UK; 3 Regional Centre for Child and Youth Mental Health and Child Welfare, Faculty of Health Sciences, UiT The Arctic University of Norway, Tromsø, Norway

**Keywords:** Alcohol use, Theory of planned behaviour, Longitudinal, Adolescents

## Abstract

**Background:**

Adolescence is a critical period for the initiation of alcohol use. It is uncertain how predictors of alcohol onset, such as Theory of Planned Behavior (TPB) constructs, change in response to alcohol onset. This study tests for longitudinal changes in TPB constructs during adolescents’ onset of alcohol consumption.

**Method:**

Data were collected longitudinally in three waves (T1, T2, T3) in Norway. Participants (*N* = 1,548; *M*_age_ = 13.54, 51% girls, 49% boys) reported alcohol use and TPB constructs (attitudes; subjective norms (SN); Perceived Behavioural Control (PBC); intentions) at each wave. Multilevel modelling was used to test whether the constructs changed over time, and intraclass correlation coefficients were used to assess the total variance explained by differences between individuals and waves. In addition, we tested different lagged versions of the TPB model, to determine whether alcohol use at wave 3 was linked to TPB constructs at earlier waves.

**Results:**

Alcohol consumption and the TPB constructs varied between waves (58% − 72%). Alcohol use, attitudes and friends-related SN significantly increased from T1 to T2 and from T1 to T3. Intentions and parent-related SN significantly increased only from T1 to T3. PBC significantly decreased only from T1 to T3. Attitudes changed the most, and PBC changed the least over time. Tests of lagged models showed that alcohol use was predicted by constructs measured at earlier waves.

**Conclusion:**

Adolescent alcohol use was mirrored by changes in attitudes and friends-related SN, whereas changes in intentions, parents-related SN and PBC took longer to manifest. In addition, constructs assessed early in adolescence retained their ability to predict alcohol use later in adolescence, although intentions measured closer to the actual behaviour showed higher accuracy in predicting alcohol use. These findings can be used to determine the optimal timing to intervene in different constructs to delay adolescent drinking onset.

**Supplementary Information:**

The online version contains supplementary material available at 10.1186/s13011-026-00726-5.

## Background

Adolescence is a time in which many changes in young people’s personal and social lives occur. A change that occurs for many is the onset of alcohol use [1, 2]; Pinquart’s meta-analysis estimated that the largest increase in alcohol consumption takes place between 12 and 13 years of age [3]. As early alcohol use onset is associated with negative health and social outcomes [4–6], it is important to identify factors that predict onset and use, and determine if these constructs remain stable during adolescence. Achieving the first goal helps identify constructs to target interventions to delay onset, and achieving the second goal helps develop an understanding of when to deliver interventions that target these constructs by mapping out when the constructs change.

Ajzen’s Theory of Planned Behaviour (TPB) is a model of motivation which has been used to predict alcohol outcomes [7]. The TPB proposes that one’s intention is the most proximal predictor of any behaviour. Intentions are predicted by three factors: Attitudes, which are positive/negative evaluations of behavioural performance; Subjective Norms (SN), which are perceptions of approval/disapproval from significant others about behavioural performance; Perceived Behavioural Control (PBC) which is a perception of control over behavioural performance. The meta-analyses by Cooke et al. and Hagger et al. provide support for several TPB relationships regarding alcohol consumption: attitudes have a large-sized relationship with intentions; SN have a medium-sized relationship with intentions; and intentions have a large-sized relationship with consumption [8, 9]. Conversely, meta-analyses revealed that PBC had an inconsistent relationship with intentions and did not correlate with alcohol use, indicating a discrepancy between perceived and actual control over drinking [10, 11].

A limitation of these meta-analyses is that because most included studies recruited participants aged 18–30 years, the results for TPB relationships may not be generalizable to adolescent samples, especially those who have not initiated alcohol use. A few studies have tested the utility of the TPB regarding alcohol outcomes in adolescent samples. Rise and Wilhelmsen reported that attitudes, SN and PBC were correlated with the intention to not drink in separate samples of Grade 7 and 9 pupils from Norway [12]. Spijkerman et al. reported that attitudes, subjective norms and PBC all predicted drinking intentions, with a negative effect for PBC, i.e., lower control predicted higher intentions, in a sample of Grade 7 and 8 pupils from the Netherlands [13]. Finally, Kyrrestad et al. used a longitudinal design to track a sample of Norwegian adolescents from 8th through 9th grade to predict intentions and onset [14]. For adolescents who had not started drinking, they reported that only SN predicted intentions, whereas the intention to drink was related to onset.

As adolescence is a time when many individuals initiate alcohol use, it is important to test the temporal stability of TPB alcohol constructs by measuring constructs longitudinally to clarify when or if they change. Hagger and Hamilton noted that few TPB studies have been conducted where constructs are measured on two or more occasions via longitudinal designs [15]. The TPB constructs are supposed to reflect the experience of behavioural performance, so the TPB constructs reported by abstinent adolescents are likely to differ from the TPB constructs reported by adolescents who have initiated alcohol use. If TPB constructs do change during adolescence in response to alcohol use onset, it would be useful from an applied perspective to determine when constructs change. For example, knowing that certain constructs can change within months, while others take years to change, could help interventionists determine which constructs to target in interventions delivered at different points of adolescence.

A related issue concerns the uniformity of changes in TPB constructs, i.e., do TPB constructs vary in terms of whether they change in the short term or long term? For example, it is conceivable that attitudes will change at an earlier point of adolescence relative to other TPB constructs. Cook et al. noted that children who have not yet initiated alcohol use hold opinions about others’ alcohol use and that attitudes towards sipping alcohol became more positive between the ages of 11 and 12 years [16]. Similarly, Leung et al. noted that having peers who consume alcohol is associated with alcohol initiation, indicating that peer approval (SN) is likely to influence adolescents’ alcohol onset [17]. As more peers initiate onset, we expect perceptions of peers’ approval to increase over time. Conversely, because perceptions of parental approval for alcohol onset are likely to be more negative, as parents are more likely to disapprove of their children drinking, values for parental SN should remain stable for a longer period of time. In contrast, intentions are likely to remain relatively stable until onset occurs, as one would expect that intentions to drink will be low prior to onset. Relatedly, PBC might remain more stable prior to onset, as it is easy to imagine perceiving high levels of control over a behaviour you are not performing.

A final issue is to determine if the predictive power of TPB constructs varies over time. Ajzen proposed that predictive power of TPB constructs is likely to decrease as the timeframe between measurement of constructs and measurement of behaviour increases; a longer timeframe increases the chance that measures of constructs no longer reflect individuals’ salient concerns [18]. While Cooke and Sheeran provide support for the idea that temporally stable constructs better predict outcomes, these studies evaluate stability over weeks and may thus not generalise to interventions where outcomes are assessed months or even years later [19–21]. The present study allows for a novel test of the idea that the predictive power of TPB constructs varies over time.

### Aim of study

The main aims of this longitudinal study were to (1) test the temporal stability of alcohol use and TPB constructs, (2) identify when TPB constructs change during adolescence, (3) explore which TPB constructs change the most during adolescence and (4) examine whether constructs measured earlier in adolescence can predict alcohol use at the end of 9th grade.

## Methods

### Participants and procedures

The current study is a part of the W8 [wait] project with data collected between 2011 and 2013 in Norway. The project aimed to evaluate the effectiveness of the universal prevention program “Youth & Alcohol” on adolescents’ alcohol use [22, 23]. The study employed a quasi-experimental longitudinal design involving 41 junior- high schools. Among these, 24 schools constituted the intervention group and were exposed to the program, whereas 17 schools formed the comparison group and followed the standard curriculum. Active written consent from parents or legal guardians was obtained for 2,020 adolescents prior to participation. Parents/guardians and their children received detailed written information about the study, and adolescents were informed that participation was voluntary and that declining to participate would carry no adverse consequences. The study was approved by the Regional Committee for Medical and Health Research Ethics (ref. nr. 2010/1082).

The analyses revealed no statistically significant differences in the rate of change between the intervention group and the control group, either for adolescents’ frequency of alcohol use or for the TPB constructs [23]. Hence, the present study includes all the responding participants as one homogeneous sample.

Adolescents were in the middle of the 8th grade (13–14 years of age) when the data were collected for the first time (T1). The post-test data (T2) were collected four months after T1, when the adolescents were at the end of 8th grade. The follow-up data (T3) were collected at the end of the 9th grade (14–15 years of age), twelve months after T2. In the present study, we included only those participants who answered at baseline (*N* = 1,548, response rate 76.6% of the 2,020 who provided parental consent). Among the 1,548 students included in this study, 1,204 answered the questionnaire at T2 (response rate: 77.7%), and 926 answered T3 (response rate: 59.8%). Participant flow and attrition analysis are detailed in Strøm et al. [23]. To evaluate potential attrition bias, we compared dropouts with completers [14]. Apart from boys being more likely to drop out, no baseline differences in alcohol use or TPB constructs were found, suggesting minimal risk of bias.

### Measures

The demographic part of the survey included information about sex (boy (1) or girl (2)) and age at baseline. The frequency of alcohol use was assessed with one item, “How often have you consumed alcohol during the past three months?”, with response categories from “No times in the past three months” (1), “1–2 times in the past 3 months” (2), “1–3 times per month” (3), and “1–7 times a week in the past three months” (4).

The TPB constructs were measured as follows (see Table 1 for all the items). Intentions were assessed via one item: “How likely is it that you will drink at least one glass of alcohol in the next 3 months?”. The response categories were rated on a five-point scale ranging from “Totally unlikely” (1) to “Totally likely” (5). Attitudes were assessed via the mean of four items (e.g., “Parties get more fun when people drink alcohol”) from the Alcohol Expectancy Questionnaire for adolescents [24, 25]. The responses were rated on a seven-point scale ranging from “Strongly disagree” (1) to “Strongly agree” (7), and the Cronbach's alpha for this scale was α = 0.74. SN were evaluated using two questions: friends-related SN: “Would your friends like or dislike it if you were drinking at least one glass of alcohol?” and parent-related SN: “Would your parents/guardians like or dislike it if you were drinking at least one glass of alcohol?” The response categories were “Disliked it strongly” (1); “Disliked it” (2); “Would not care” (3); and “Like it a little/very much” (4). Since those two items were only moderately correlated (Pearson correlation = .33), they were considered separately (i.e., not as a scale) in the analyses. PBC was assessed via the mean of four items (e.g., “It will be difficult/easy for me not to drink any alcohol for the next 3 months”; see Table 1 for all included items), which were rated on a seven-point scale ranging from “Very difficult” (1) to “Very easy” (7) for items 1 to 3 and from “I do not know any” (1) to “Yes, I know several” (7) for item 4. Higher PBC scores thus indicate greater perceived ease of abstaining from alcohol. Cronbach's alpha for this scale was α = 0.77. 

**Table 1:** Descriptive statistics of the items used to construct multilevel models

		T1			T2			T3		
		*N*	Mean (SD)	CV	*N*	Mean (SD)	CV	*N*	Mean (SD)	CV
***Alc. Use*** - Overall Mean (*SD*): 0.22 (0.59)										
Alc	How often have you consumed alcohol during the past three months?	1543	1.12 (0.42)	0.38	1150	1.15 (0.48)	0.42	890	1.49 (0.83)	0.56
***Int*** - Overall Mean (*SD*): 1.77 (1.22)										
Int	How likely is it that you will drink at least one glass of alcohol in the next 3 months?	1539	1.62 (1.13)	0.69	1192	1.66 (1.12)	0.67	906	2.17 (1.40)	0.64
***SN*** - Overall Mean (*SD*) SN-1: 2.30 (0.83), SN-2: 1.30 (0.53)										
SN-1	Would your friends like or dislike if you were drinking at least one glass of alcohol?	1540	2.16 (0.82)	0.38	1196	2.25 (0.82)	0.36	920	2.60 (0.79)	0.30
SN-2	Would your parents/guardians like or dislike if you were drinking at least one glass of alcohol?	1541	1.26 (0.50)	0.40	1203	1.27 (0.51)	0.40	919	1.41 (0.59)	0.42
***Mean PBC*** - Overall Mean (*SD*): 5.87 (1.26)		1545	5.92 (1.29)	0.22	1198	5.87 (1.21)	0.21	912	5.76 (1.26)	0.22
PBC-1	I think it will be easy/difficult to say no to a glass of beer or wine in a party with classmates if one person I like drinks and offers me a glass	1521	5.68 (1.74)		1187	5.58 (1.71)		907	5.48 (1.84)	
PBC-2	If I get offered some alcohol, I think it is difficult/easy to say no thanks	1516	6.04 (1.56)		1186	5.98 (1.48)		906	5.89 (1.59)	
PBC-3	It will be difficult/easy for me not to drink any alcohol for the next 3 months	1509	6.38 (1.55)		1187	6.33 (1.49)		903	5.99 (1.72)	
PBC-4	If someone offers me a glass of beer or wine, I know/do not know some good ways to say no	1538	5.62 (1.69)		1194	5.63 (1.59)		910	5.68 (1.62)	
***Mean Att*** - Overall Mean (*SD*): 2.74 (1.42)		1535	2.47 (1.28)	0.52	1188	2.67 (1.37)	0.51	898	3.30 (1.57)	0.48
Att-1	Adolescents are happy and have a good time when they drink alcohol	1532	2.64 (1.70)		1185	2.86 (1.75)		892	3.56 (1.82)	
Att-2	It is OK to drink alcohol, because one can then get to be with other adolescents who are having fun	1520	1.75 (1.33)		1179	2.03 (1.47)		889	2.66 (1.81)	
Att-3	Parties get more fun when people drink alcohol	1507	2.83 (1.89)		1171	2.88 (1.89)		883	3.61 (2.02)	
Att-4	Many alcoholic beverages taste good	1509	2.66 (1.83)		1159	2.89 (1.90)		880	3.38 (2.04)	

Note: Measures are made at three timepoints through adolescence (T1, T2 and T3). SD = Standard Deviation; CV = Coefficient of Variation; Alc. use = Alcohol use (from “No times in the past three months” (1) to “1-7 times a week in the past three months” (4)); Int = Intentions rated from “Totally unlikely” (1) to “Totally likely” (5); SN = Subjective Norms (from “Disliked it strongly (1) to “Like it a little/very much” (4)); PBC = Perceived Behavioral Control (from “Very difficult” (1) to “Very easy” (7) for items 1 to 3, and from “I do not know any” (1) to “Yes, I know several” (7) for item 4)); Att = Attitudes (from “Strongly disagree” (1) to “Strongly agree” (7))

### Statistical analyses

The data were analysed via IBM SPSS Version 29 and Mplus Version 7.4. To ensure the TPB constructs were comparable over time, we tested for longitudinal measurement invariance of the multi-item constructs (i.e., Attitude and PBC) across the three waves. This procedure is essential to confirm that the mean scores used in our analyses represent the same underlying psychological concepts at each wave. For that, we compared a configural model (where the relationships between the individual items and the overall construct were allowed to vary freely between waves) to a more restrictive metric model (where these relationships were constrained to equality across T1, T2, and T3). Given the sensitivity of the Chi-square test to sample size, invariance was evaluated based on a change in the Comparative Fit Index (ΔCFI) of less than 0.010, as recommended by Cheung and Rensvold [26].

The means and standard deviations (*SDs*) for the study variables were calculated along with the coefficients of variation (*CVs*), also known as the relative standard deviation, which is calculated by dividing the standard deviation by the mean (see Table 1). CVs are a relative measure of dispersion that can be used to compare the variations in different variables. We used CVs to compare variability between different constructs at a given timepoint and not between waves since the intraclass correlation coefficients (ICCs) obtained via multilevel modelling were used to evaluate variability between waves (see below).

We used multilevel models to run our analyses since we had repeated measures from the same individuals, and consecutive answers given by the same individual were not totally independent from one another. We first ran models with just an intercept and an identity covariance structure for the random effects to estimate ICCs for each variable. In repeated measures designs, the ICC can be used to evaluate how much of the overall variation in the outcome is explained by differences between waves vs. differences between individuals. ICCs can help to assess the extent to which the TPB constructs remain stable for each individual between waves or vary between waves but remain stable between individuals.

We compared different covariance structures for the random effects and found that the diagonal covariance structure, which allows for heterogeneous variances and zero correlation between elements, was the one that fit the data best for all variables. Thus, we used this covariance structure to test for longitudinal changes in the TPB constructs and included the variable *time* (coded as a categorical variable T1, T2, T3) in all the models. We ran models on both unstandardized (i.e., variables that retain their original scaling) and standardized variables (i.e., variables that have been rescaled to have a mean of zero and a standard deviation of one) to obtain both types of regression coefficients. Unstandardized coefficients are easier to interpret since they represent the relationship between a dependent variable and the independent variables on their original scales. They can be used in calculations and to examine how the dependent variable changes when a given independent variable changes with one unit. In multiple regressions, however, unstandardized coefficients cannot be used to compare the effects of different independent variables when the measurement scales of the independent variables are different. In such multiple regressions, the standardized regression coefficients represent the expected change in the number of standard deviations of the dependent variable when one of the independent variables increases by one *SD* while the other dependent variables remain constant and can be used for comparing the effects of the different independent variables on the dependent variable.

In our analyses, we had only one categorical independent variable in each model (*time*), so the standardized coefficients represent the change in the number of *SDs* of the dependent variables from T1 to T2 or from T1 to T3. Consequently, we do not use standardized regression coefficients to compare the effects of different independent variables on the same dependent variable but rather to compare the effects of the independent variable *time* between models. This helped us determine which of the TPB constructs changed the most over time.

In addition, we used structural equation models (SEMs) with lagged constructs to investigate whether TPB constructs measured earlier in adolescence can predict alcohol use at the end of 9th grade (T3). We used the procedures described in Kyrrestad et al.to build multilevel SEM models where the class attended by the student was used as cluster variable [14]. The only exception to these procedures is that we model here the subjective norms as two separate observed variables instead of two items loading on a single SN-latent variable. This allows to distinguish between peer and parental influences, which often diverge during adolescence, providing a more precise estimation of their independent effects on drinking intentions. We labelled the examined models according to the timepoints when the different variables were measured. For example, the model labelled T1-T2-T3 uses attitudes, SN and PBC measured at T1; intention measured at T2; and alcohol consumption measured at T3. This is the same base model as the one used in our former study and we call it the “TPB by time” model [14]. Other models evaluated include for example the “cross-sectional” model, where all variables are measured at T3 and the “shorter-term prediction” model, where constructs measured at T2 (i.e., 4 months before T3) are used to predict alcohol use at T3.

## Results

### Descriptive statistics

**Fig. 1:**
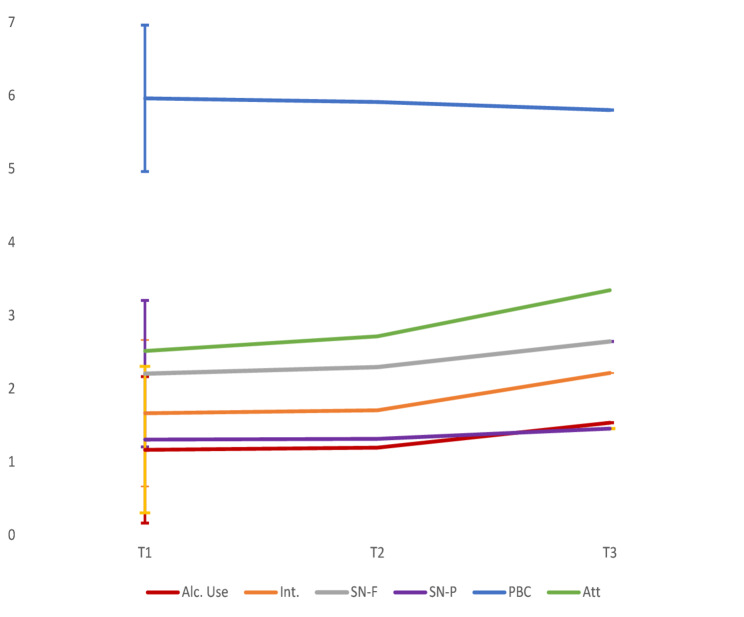
Time trend for the TPB factors. Mean predicted scores from multilevel models for alcohol use, intention, attitudes, SN, and PBC. *Note*: Alc. use = Alcohol use (from “No times in the past three months” (1) to “1-7 times a week in the past three months” (4)); Int = Intentions rated from “Totally unlikely” (1) to “Totally likely” (5); SN - F = Subjective Norms Friends and SN - P = Subjective Norms Parents (from “Disliked it strongly (1) to “Like it a little/very much” (4)); PBC = Perceived Behavioral Control (from “Very difficult” (1) to “Very easy” (7) for items 1 to 3, and from “I do not know any” (1) to “Yes, I know several” (7) for item 4)); Att = Attitudes (from “Strongly disagree” (1) to “Strongly agree” (7))

Overall, 1,548 adolescents with a mean age of 13.54 years (*SD* = 0.31) at baseline and a balanced gender distribution (51% girls and 49% boys) participated in the study. At T1, 23.5% of the adolescents reported having onset drinking (i.e., having drunk at least one glass of alcohol in their life), whereas this percentage had increased to 48.5% at T3. For the frequency of alcohol consumption, 8.9% reported at T1 that they had been drinking at least one glass of alcohol in the past three months, whereas this percentage had increased to 30.0% at T3. Table 1 shows the means and *SDs* for alcohol use and the TPB constructs at each wave. For an illustration of the time trends, see Fig. 1. Measurement invariance analyses supported the stability of the TPB constructs over the 16-month study period. The drop in CFI between the configural model (CFI = 0.980) and the metric model (CFI = 0.976) was well below the recommended 0.010 threshold.

The mean attitudes scores showed the greatest increase from T1 to T3, whereas the SN and intentions scores showed moderate increases from T1 to T3; however, there were slightly greater changes in friend-related SN than in parent-related SN. In contrast, PBC showed evidence of a slight decrease from T1 to T3 (with lower scores indicating more difficulty abstaining), although this was the most stable of the constructs (Table 1). The CVs revealed that intentions and attitudes were the constructs for which we observed the most variability between individuals at T1 and T2, whereas intentions and alcohol use were the factors with the most variability at T3 (Table 1).

### Variance in the TPB constructs between individuals and between waves

The ICCs revealed that alcohol use varied greatly between waves (72% of the variance) and relatively little between individuals (28%). A similar pattern occurred for the TPB constructs: intentions and PBC also varied more between waves (66% and 64% of the variance, respectively), as did SN (61% and 64% of the variance for SN friends and SN parents, respectively) and attitudes (58% of the variance). Nevertheless, attitudes and friend-related SN were the TPB constructs that varied the most between individuals (39–42% of the variance lied between individuals for those variables vs. approximately 34–36% for the other constructs).

### Longitudinal changes in alcohol use and the TPB constructs

Mixed models testing the effect of *time* on adolescents’ alcohol use and the TPB constructs revealed statistically significant effects of time in all the models (Table 2). The frequency of alcohol use was greater at T2 than at T1 and even greater at T3 than at T1, indicating that the frequency of alcohol use increased gradually during adolescence. Similar patterns were found for attitudes and friends-related SN, which increased significantly both between T1 and T2 and between T1 and T3. In contrast, parent-related SN and intentions increased significantly between T1 and T3, and PBC decreased significantly between T1 and T3; however, none of these constructs significantly changed between T1 and T2.

**Table 2:** Unstandardized and standardized results for multilevel models examining changes in the TPB constructs through adolescence

	Unstandardized coefficients			Standardized coefficients		
	Intercept	T2	T3	Intercept	T2	T3
Alcohol use	1.121 (0.011)***	0.044 (0.014)**	0.369 (0.026)***	− 0.171 (0.019)***	0.075 (0.024)**	0.627 (0.044)***
Intentions	1.625 (0.029)***	0.056 (0.033)	0.565 (0.047)***	− 0.121 (0.024)***	0.046 (0.027)	0.463 (0.038)***
Attitudes	2.468 (0.033)***	0.213 (0.036)***	0.867 (0.048)***	− 0.191 (0.023)***	0.149 (0.026)***	0.608 (0.034)***
PBC	5.920 (0.033)***	− 0.053 (0.038)	− 0.185 (0.045)***	0.043 (0.026)	− 0.042 (0.031)	− 0.147 (0.036)***
SN-Friends	2.165 (0.021)***	0.091 (0.024)***	0.447 (0.027)***	− 0.164 (0.025)***	0.110 (0.029)***	0.537 (0.032)***
SN-Parents	1.260 (0.013)***	0.011 (0.015)	0.149 (0.020)***	− 0.077 (0.024)**	0.022 (0.028)	0.278 (0.037)***

Note: **p < .01, ***p < .001. Measurements at three timepoints: T1, T2 and T3. Timepoint 1 is the reference category for fixed effects. PBC = Perceived Behavioral Control; SN = Subjective Norms

A comparison of the standardized coefficients between the models, presented in Table 2, revealed that attitudes were the TPB construct that changed the most between T1 and T2, since it increased by 0.149 standard deviations, whereas the other constructs changed by only between 0.042 and 0.110 standard deviations during this period. Between T1 and T3, attitudes were again the construct that changed the most (0.608 standard deviations), and alcohol use also increased greatly during that period (by 0.627 standard deviations). Across both periods, PBC was the most stable construct, which decreased by only 0.042 and 0.147 standard deviations, respectively.

### Predictive power of the TPB constructs over time

The TPB models built using lagged constructs are presented in Table 3 and Appendix 1. The “cross-sectional” model (i.e., where all variables are measured at T3) produced a poor model fit (CFI<0.95 and RMSEA>0.05, Table 3) although it did explain the most variance in alcohol use (*R*^2^ = 0.72) and in intention to drink (*R*^2^ = 0.53). It was also the only model where the association between PBC and alcohol use was positive, and where the factor loading for the PBC4 item on PBC was rather low (0.37 ± 0.036, see Appendix 1). This might be due to the items used in this model being highly correlated to one another and thus generating high *R*^2^, but untrustworthy parameter estimates.

**Table 3:** The TPB-models over different timepoints

	Chi^2^	CFI	RMSEA	R^2^ Alcohol	R^2^ Intention
“Cross-sectional” (T3-T3-T3)	411.3	0.922	0.093	0.72	0.53
“Shorter-term prediction” (T2-T2-T3)	161.7	0.979	0.043	0.30	0.26
“Longer-term prediction” (T1-T1-T3)	167.4	0.983	0.041	0.16	0.24
“TPB by time” (T1-T2-T3)	136.2	0.987	0.036	0.26	0.19
“Stable beliefs” (T1-T3-T3)	117.8	0.989	0.032	0.54	0.21
“Updated beliefs” (T2-T3-T3)	104.1	0.989	0.030	0.60	0.23

Note: Model fit indices (Chi-square: Chi^2^, Comparative Fit Index: CFI, Root Mean Square Error of Approximation: RMSEA), and proportion of variance explained in alcohol use (R^2^ Alcohol) and in intention to drink (R^2^ Intention) in the 6 TPB models examined. Models are built using different timepoints for the different constructs. Timepoints are given in the following order: Attitude/SN/PBC- Intention- Alcohol use)

The results indicate a clear temporal attenuation of the intention-behavior relationship. The variance explained in alcohol consumption (R^2^ Alcohol) decreased significantly as the time gap between intention and behavior measurements increased. When intention and alcohol use were measured concurrently at T3 (e.g., in the updated beliefs model), the model accounted for up to 60% of the variance in behavior. However, this predictive power dropped to 30% in the shorter-term prediction model (4-month gap) and further to 16% in the longer-term prediction model (16-month gap).

In contrast, the prediction of intention remained notably stable across different time points. All models except the cross-sectional model accounted for about 20–25% of the variance in intentions, suggesting similar predictive power of attitudes, SN and PBC measured either at T1 or at T2.

## Discussion

This study aimed to test the stability of alcohol use and of the TPB constructs, to identify when and which of the TPB constructs change the most during adolescence and to examine whether the predictive power of TPB constructs varies over time. Our results show that as adolescents’ alcohol use increased during adolescence, as is expected in a sample of this age group, their attitudes, SNs, and intentions also increased, whereas their PBC decreased, suggesting lower perceived control. Changes in constructs seem to occur at different timepoints; however, the changes in attitudes and friends-related SN mirrored the changes in alcohol use, whereas parent-related SN, intentions and PBC changed significantly only over a longer period of time (i.e., 16 months after the baseline measures were collected), and PBC decreased rather than increased, similar to the other TPB constructs. Additionally, while intentions and attitudes were the constructs that varied the most between students at baseline, alcohol use became one of the most variable factors towards the end of the study, probably because alcohol use increased for some but not all individuals. Finally, we found that the predictive power of TPB constructs did vary over time, although constructs measured early in adolescence could predict alcohol use at the end of 9th grade.

This study provides a longitudinal test of the idea that TPB constructs regarding alcohol use change over time. This novel finding highlights the benefit of using a longitudinal design to evaluate how constructs change over time, with the additional benefit of examining change in these constructs during adolescence, a period when behavioural changes, especially those related to alcohol, occur. A common criticism of TPB studies is that few adopt longitudinal approaches to test the prediction of behaviour over time and assess the extent of change within constructs during periods of change in participants’ lives. Previous research on the temporal stability of TPB constructs has shown that more stable constructs better predict outcomes [19]. However, such studies are typically conducted with young adult samples, who are not experiencing the developmental changes found in adolescence. This study adds to the literature by showing what happens to TPB constructs during more changeable times in individuals’ lives.

Hagger and Hamilton’s recent meta-analysis of TPB studies using longitudinal designs provides an overview of studies testing the TPB longitudinally and highlights the pitfalls of only measuring TPB constructs at baseline and following-up participants within a prospective timeframe of approximately six weeks or less [15]. If we had measured the TPB constructs six weeks after baseline, we would have missed important changes in the TPB constructs. For example, parent-related SN, intentions and PBC only showed a statistically significant change sixteen months after baseline measures, which helps make sense of the observed increase in alcohol use and informs our thinking about theoretical predictions. It is important researchers ensure that their study designs can capture important changes in constructs by matching designs to patterns of behaviour performed by their samples. Doing this will allow them to draw firmer conclusions from their data.

A further advantage of using a design where TPB constructs and outcomes were measured at three timepoints is we were able to test the idea that predictive power of TPB constructs varies over time. While our “cross-sectional” model accounted for the most variance, it had poor model fit indices, indicating overlap between measures. In contrast, tests of models where constructs were measured at earlier timepoints showed better fit to the data and confirm that larger timeframes between measurement do affect prediction of behaviour. The “shorter-term prediction” and “TPB by time” models, which both used T2 measures of intentions to predict alcohol use at T3, accounted for similar variance in use, whereas the “Longer-term prediction” model, which used T1 measures of intentions, accounted for lower variance in alcohol use. Such results provide additional support for Ajzen’s claim that larger timeframes between measurement of constructs and outcomes can reduce predictive power [18]. However, results for intentions were less consistent with this claim; we found that all models accounted for similar amounts of variance in intentions, although the lowest value was for the “TPB by time” model, i.e., the one with a 4-months gap between measurement points. Nevertheless, a model that predicts intentions four months later (i.e., the TPB by time model) which explains similar levels of variance to models predicting intentions cross-sectionally (e.g., in the “Shorter-term prediction” or the “Longer-term prediction” models), suggests that while predictive power reduces over time it does not disappear entirely. Given most interventions typically use larger gaps between measurement points than studies testing effects of TPB models, it is encouraging that results show these constructs retain predictive power over time. Overall, we believe our analyses provide a realistic estimate of how much variance in outcomes can be expected to be accounted for when constructs are measured months/years earlier. These estimates can help future interventionists determine issues of study design, when to measure constructs and outcomes, alongside thinking about changes in outcomes.

### Implications for theory

Although there have been calls to retire the TPB [27], our findings are consistent with the rebuttals of this call [28] because they show that the TPB can be a useful model when accounting for alcohol use. For instance, an important finding from our analyses is that while alcohol use and TPB constructs changed over time, there was variation in the extent, and direction, of change for the different constructs. For example, both attitudes towards alcohol use and friend-related SN increased more from T1 to T3 than did parent-SN and intentions, whereas PBC decreased over time. These results suggest that evaluations of alcohol use and perceptions of friends’ approval of alcohol use are more sensitive to increases in alcohol use than parental disapproval of, or perceptions of, control over, alcohol use. The fact that attitudes increase the most over time can reflect several things.

First, average attitudes towards alcohol at baseline were below the midpoint of the scale, suggesting mildly negative evaluations of drinking. Sixteen months after T3, average attitudes approached the midpoint of the scale, and it is possible that if we had followed our sample at later ages, their attitudes would have increased further. This pattern is supported by the findings of Kyrrestad et al., who reported that as adolescents gain more experience with alcohol drinking, their attitudes towards alcohol become increasingly positive [14]. Second, there is evidence that evaluations of alcohol use develop prior to other cognitions. Cook and colleagues reviewed the literature on young children’s (i.e., aged 2–12) perceptions of alcohol use by others and noted that even young children hold opinions about others’ (parents, family members) alcohol use [16]. Therefore, it is possible that attitudes towards alcohol develop earlier than other TPB constructs do, which may rely more on experiential processes.

It is also possible that prior to alcohol use onset, adolescents perceive widespread disapproval of alcohol use, leading to changes in norms when peers’ alcohol consumption becomes more evident. Adolescents’ alcohol consumption has been shown to be highly influenced by their own belief (SN) of how their family and friends would like them to behave [14]. Similarly, perceptions of control over drinking are unlikely to develop until alcohol use onset, since there is not much need or opportunity to perceive control over drinking until one has started. The mean values for PBC measured in this study are very high, approaching the ceiling of the scale, which makes sense, as it is easy to perceive PBC as being in control before alcohol consumption begins. Our findings at T3 show that PBC has not decreased substantially by the end of 9th grade, suggesting that alcohol use patterns are not (yet) associated with the lack of control we typically find reported by older adolescents and young adults [8]. Two previous studies examining PBC in adolescents via cross-sectional designs reported either a positive correlation between PBC and intentions [12] when individuals were asked about their control over avoiding drinking and intentions to not drink or a negative correlation between PBC to refrain from drinking and the intention to drink [13], suggesting that the results are sensitive to what action (to drink or refrain from drinking) participants were surveyed about. The advantage of using a longitudinal design is to demonstrate the change in PBC during adolescence (i.e., over a period of 16 months); our findings indicate a gradual decrease in PBC as alcohol use among adolescents increases. This trend suggests that as adolescents age, they are increasingly exposed to a more supportive normative environment for alcohol use and increasingly positive attitudes towards drinking. Consequently, this could make it more challenging for them to decline friends’ offers of alcohol and to decrease their alcohol consumption over time. The current results are consistent with the idea that a lack of control is linked to increased consumption over time.

### Implications for practice

Because the W8 [wait] project used a longitudinal design and collected data at several waves during adolescence, we were able to examine how the TPB constructs changed over a longer timeframe than is often possible. We encourage all interventionists to consider which theoretical questions could be addressed alongside their primary goal of evaluating changes in outcomes. This approach can represent a win‒win for funders and research teams; funders obtain more outputs in return for their investment, whereas researchers better understand what happens to mediator variables during their intervention. To make this situation a reality, research teams need to ensure that these secondary outcomes (e.g., attitudes, SN) are measured on multiple occasions.

Our finding that some constructs changed earlier than others (i.e., within 4 months vs. within 16 months) have implications for the timing of interventions. For example, since attitudes and friends-related SN change the fastest, it may be more efficient to target these constructs when interventions are designed for the youngest adolescents. In contrast, focusing on intentions might be more appropriate for older adolescents. In addition, our results also show that constructs measured earlier in adolescence retain their predictive power on the intention to drink measured 4 months later, suggesting that addressing TPB constructs early may have a lasting impact. Nevertheless, interventionists should be aware that attempting to make constructs more negative may not be feasible during periods of change in adolescents’ lives. For example, in a small-scale pilot study [29], it was possible to maintain the level of attitudes towards alcohol in an intervention group compared with a control group whose attitudes increased over time but not to reduce those attitudes. Interventionists need to be mindful of what is feasible during different periods of participants’ lives and consider the context surrounding participants when trying to change behaviour.

From a health-promotion perspective, early targeted efforts could, for example, reinforce adolescents’ perceived control before it diminishes with increased alcohol use. By recognizing the individual variability in behaviour change, health-promotion programs can also be tailored to meet adolescents’ needs. For example, we could survey younger adolescents to measure their alcohol use onset and TPB constructs to develop a profile of their alcohol use and beliefs about alcohol and then deliver intervention materials that address issues with beliefs; for some individuals, it might be that attitudes need to be challenged, whereas for others, their perceptions of peers’ normative approval need to be challenged. In a cost-effective manner, these interventions could use digital platforms to deliver personalized interventions, which reduces costs compared with traditional methods. This approach promises more effective support for young people in making healthier choices and fostering resilience during these formative years.

### Strengths and limitations

A key strength of this study relates to its longitudinal design, which allowed us to assess how the TPB constructs changed over time. Additionally, the study was conducted among adolescents at a time when many of them began to drink alcohol, which means that the theme was current and that the implications for practice can be especially relevant for preventing the early onset of alcohol consumption. Additionally, with a large sample size and a high response rate, our findings can be considered representative of junior high school students in Norway.

Nonetheless, the findings from the current study should also be considered in the context of the study’s limitations. First, the data collection occurred between January 2011 and May 2012. While the data might be considered outdated, we argue that the data still significantly enhance our understanding of adolescent alcohol use. Furthermore, the national youth survey Ungdata in Norway indicates that the proportion of junior high school students, aged 13–16 years, who consume alcohol at least once a month has remained remarkably stable from 2010 to 2023-24 [30]. According to the Ungdata survey, in 2010, 8% of boys and 7% of girls reported monthly alcohol use, whereas by 2023-24, the percentage for both genders had stabilized at 7%. This consistency over time underscores the relevance and applicability of our findings despite the age of the dataset. Second, although our study benefited from a large sample size and high response rate, which make our findings representative of the study population, our data collection was performed only in Oslo and the neighbouring county Akershus, which means that our results may not be generalizable across other settings in other areas of Norway. Third, the validity of self-reports from adolescents in that age group should be considered. Indeed, when adolescents in our study were under the legal drinking age, they might have underreported their drinking due to a fear of unknown consequences. Conversely, as adolescents are also highly influenced by their beliefs about how their friends would like them to behave, they might also overreport their drinking due to a socially desirable bias. While other sources of information, such as qualitative interviews with adolescents or information collected from their parents and guardians, might be useful, the primary assessment form for alcohol use is individual self-reports [31], which are likely the best and most reliable source of information when dealing with adolescent samples. Fourth, we used single-item measures of intention, subjective norms, and alcohol use. This decision was taken to ease the implementation of a shorter questionnaire during school hours, as well as to minimize student burden and reduce the risk of sample attrition. A further limitation concerns the measurement of PBC. Although PBC is conceptualised as comprising both self‑efficacy and controllability [32], our measure captured only the controllability component. The scale combined items assessing perceived difficulty of abstaining from alcohol with items assessing knowledge of alcohol‑related opportunities, which represent related but distinct facets of control. Averaging these items may therefore not fully reflect a unified PBC construct and may have restricted our ability to capture the complete influence of PBC on adolescent drinking behaviour. Finally, while our analysis reveals a certain variability in the extent to which different constructs change over time, these findings need to be replicated in future studies.

## Conclusion

The results from the current study show a lack of uniformity in the timing and direction of when the TPB constructs regarding alcohol changed during adolescents’ alcohol onset. Attitudes and friend-related SN increased earlier than parent-related SN and intentions did, whereas PBC decreased over time and was clearly the most stable TPB construct within this dataset. The results from this analysis can be used to increase the understanding of theoretical predictors of adolescent alcohol use and may be beneficial in suggesting when to intervene and which constructs to intervene in when trying to delay the onset of alcohol use.

## Supplementary Information

Below is the link to the electronic supplementary material.

Supplementary Material 1

## Data Availability

The dataset used and analyzed during the current study is available from the corresponding author upon reasonable request.

## Abbreviations

ATT: Attitude
CV: Coefficients of variation
ICC: Intraclass correlation coefficients
INT: Intention
M: Mean
PBC: Perceived Behavioural Control
RMSEA: Root Mean Square Error of Approximation
SD: Standard deviation
SN: Subjective norms
TPB: Theory of Planned Behaviour
