# Supplementary material for: Do theory of planned behaviour constructs change during adolescent alcohol use onset?
Source: Subst Abuse Treat Prev Policy. 2026 Apr 10;21:37. doi: 10.1186/s13011-026-00726-5 (PMC13181889; doi:10.1186/s13011-026-00726-5)
Supplement: Supplementary file 1 — Supplementary Material 1 [file 13011_2026_726_MOESM1_ESM.docx]

**Appendix 1**: Standardized parameter estimates ± SE from the multilevel structural equation modeling of the TPB models examining frequency of alcohol use at T3 predicted by TPB constructs measured at T1, T2 or T3.

|  | “Cross-sectional” (T3-T3-T3) | “Shorter-term prediction”  (T2-T2-T3) | “Longer-term prediction” (T1-T1-T3) | “TPB by time” (T1-T2-T3) | “Stable beliefs” (T1-T3-T3) | “Updated beliefs” (T2-T3-T3) |
| --- | --- | --- | --- | --- | --- | --- |
| **Measurement part** |  |  |  |  |  |  |
| Att1-Attitude | .71 ± .019*** | .66 ± .019*** | .62 ±.018*** | .62 ±.018*** | .62 ±.018*** | .67 ± .019*** |
| Att2-Attitude | .88 ± .014*** | .85 ± .015*** | .86 ±.020*** | .86 ±.020*** | .86 ±.020*** | .85 ± .015*** |
| Att3-Attitude | .86 ± .015*** | .83 ± .013*** | .77 ±.015*** | .77 ±.015*** | .77 ±.016*** | .83 ± .014*** |
| Att4-Attitude | .73 ± .020*** | .63 ± .021*** | .67 ±.019*** | .66 ±.020*** | .66 ±.020*** | .63 ± .022*** |
| PBC1-PBC | .75 ± .019*** | .82 ± .017*** | .83 ±.014*** | .83 ±.014*** | .83 ±.014*** | .83 ± .017*** |
| PBC2-PBC | .89 ± .017*** | .93 ± .016*** | .94 ±.012*** | .94 ±.012*** | .94 ±.012*** | .93 ± .016*** |
| PBC3-PBC | .86 ± .020*** | .72 ± .023*** | .76 ±.020*** | .76 ±.020*** | .76 ±.020*** | .72 ± .023*** |
| PBC4-PBC | .37 ± .036*** | .55 ± .025*** | .53 ±.026*** | .53 ±.026*** | .53 ±.026*** | .55 ± .025*** |
| **Structural part** |  |  |  |  |  |  |
| Attitude-Intention | .45 ± .040*** | .27 ± .041*** | .29 ±.038*** | .24 ± .043*** | .33 ± .050*** | .30 ± .051*** |
| SN-Friends-Intention | .10 ± .034 ** | .17 ± .028*** | .13 ±.021*** | .15 ± .028*** | .07 ± .036 NS | .14 ± .037*** |
| SN-Parents-Intention | .08 ± .024 ** | .11 ± .025*** | .12 ±.022*** | .08 ± .027 ** | .03 ± .032 NS | .11 ± .029*** |
| PBC-Intention | -.29 ± .039*** | -.15 ± .042*** | -.13 ±.030*** | -.10 ± .034 ** | -.14 ± .044 ** | -.08 ± .048 NS |
| Intention-Alcohol Use | .94 ± .054*** | .52 ± .038*** | .33 ±.038*** | .46 ± .034*** | .74 ± .036*** | .77 ± .036*** |
| PBC- Alcohol Use | .20 ± .056*** | -.07 ± .042 NS | -.15 ±.042*** | -.12 ± .042 ** | -.02 ± .035 NS | -.01± .035 NS |

*Note*. Timepoints at which the different variables are given in the following order: Attitude/SN/PBC- Intention- Alcohol use) with T1 = Baseline; T2 = 4 months after T1; T3 =12 months after T2/16 months after T1. For example, the cross-sectional model examines all variables measured at T3, while the longer-term prediction model examines alcohol use measured at T3, but other TPB constructs measured at T1.
